# Supplementary material for: Cell-based optimization and characterization of genetically encoded location-based biosensors for Cdc42 or Rac activity
Source: J Cell Sci. 2023 May 25;136(10):jcs260802. doi: 10.1242/jcs.260802 (PMC10234108; doi:10.1242/jcs.260802)
Supplement: Supplementary information [file joces-136-260802-s1.pdf]

A

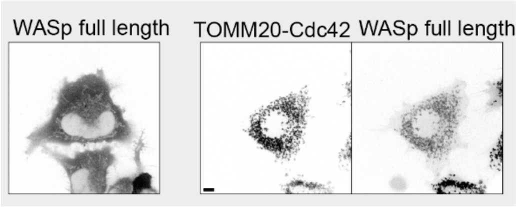

Fig. S1. (A) Spinning disk images of HeLa cells expressing mScarlet-I-WASp(full length) (left) and co-expressing of TOMM20-mTurquoise2-Cdc42(G12V)-ΔCaaX (middle) and mScarlet-I-WASp(full length) (right). Scale bar: 10 µm.

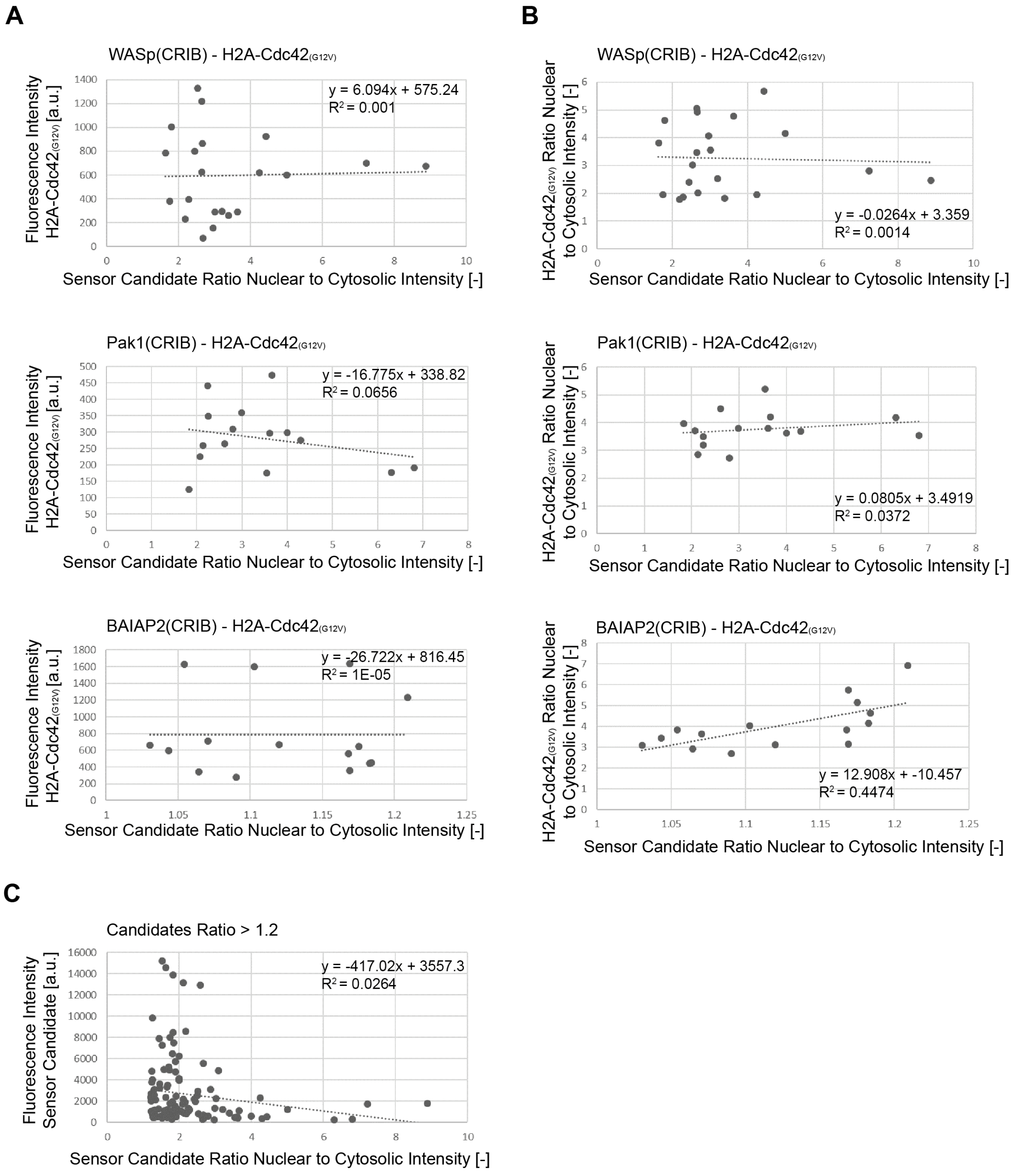

Fig. S2. (A) Plots of the correlation between expression level of H2A-Cdc42(G12V)-ΔCaaX, represented by fluorescence intensity, and sensor candidate nuclear to cytosolic intensity, at the example of WASp(CRIB), Pak1(CRIB) and BAIAP2(CRIB), for the data shown in Fig. 3B. The general linear model (top, right corner) was fit to the data and is indicated as a dashed line. (B) Plots of the correlation between H2A-Cdc42(G12V)-ΔCaaX nuclear to cytosolic intensity and sensor candidate nuclear to cytosolic intensity, at the example of WASp(CRIB), Pak1(CRIB) and BAIAP2(CRIB), for the data shown in Fig. 3B. The general linear model (bottom, right corner) was fit to the data and is indicated as a dashed line. (C) Plots of the correlation between expression level of the sensor candidate, represented by fluorescence intensity, and sensor candidate nuclear to cytosolic intensity, for all candidates with a ratio above 1.2, for the data shown in Fig. 3B. The general linear model (top, right corner) was fit to the data and is indicated as a dashed line.

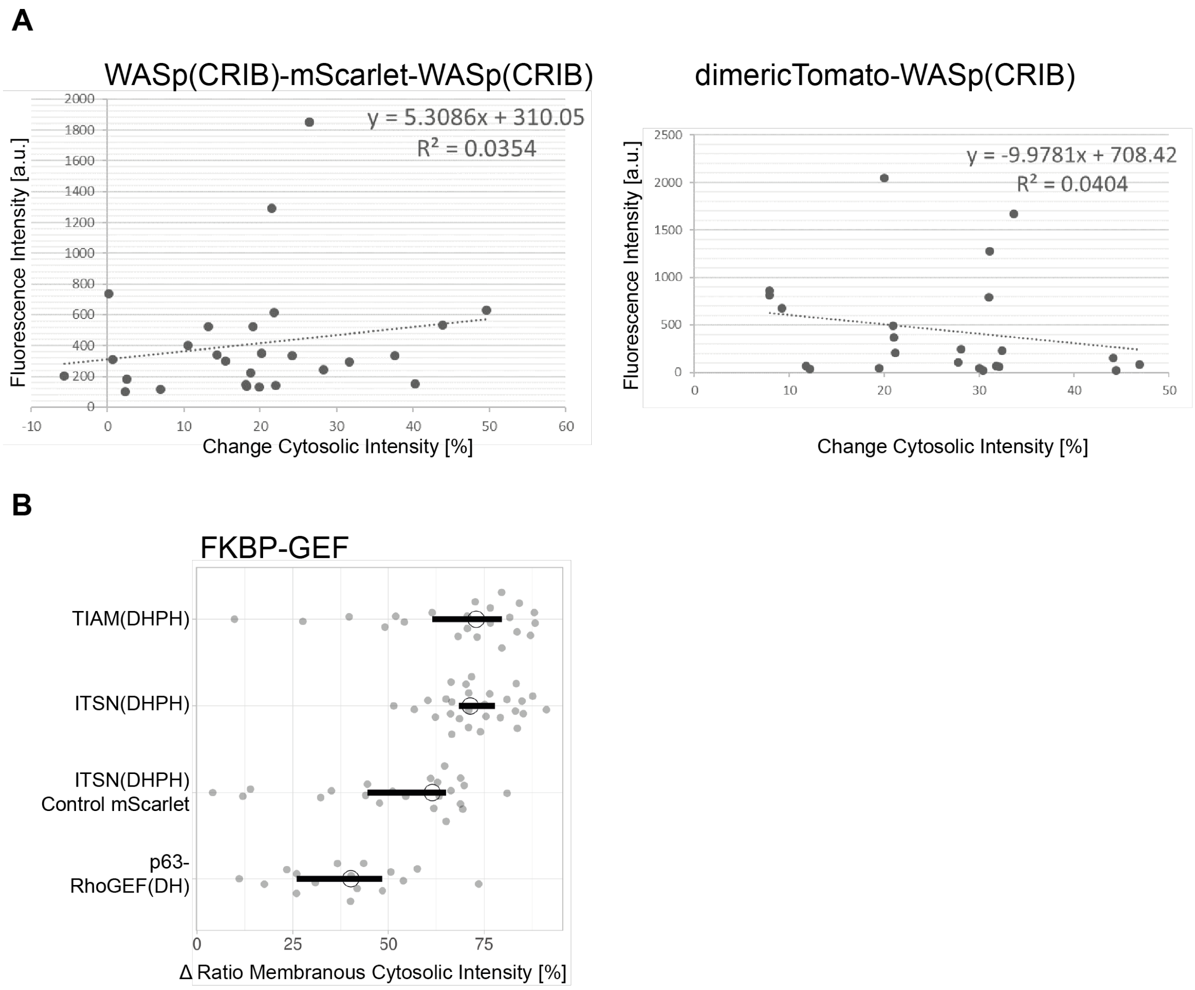

Fig. S3. (A) Plots of the correlation between fluorescence intensity and change in cytosolic intensity for cell expressing WASp(CRIB)-mScarlet-WASp(CRIB) or dimericTomato-WASp(CRIB) for the data shown in Fig. 5D,E. The general linear model (top right corner) was fit to the data and is indicated as a dashed line. (B) Relocation of the FKBP-GEF fusion protein represented by the change in ratio of membranous over cytosolic intensity measured in HeLa cells expressing Lck-FRB-mTurquoise2, dimericTomato-WASp(CRIB) or mScarlet-I as a control and either YFP-FKBP-ITSN1(DHPH), YFP-FKBP-TIAM1(DHPH) or YFP-FKBP-RhoGEFp63(DH). FKBP-GEF recruitment was stimulated with 100 nM rapamycin. The median is represented as a circle, the 95% confidence interval as a black line and each dot represents the measurement of a single cell. The number of cells measured in two experiments based on independent transfections is: mScarlet-I YFP-FKBP-ITSN1(DHPH) control=22, YFP-FKBP-TIAM1(DHPH)=22, YFP-FKBP-RhoGEFp63(DH)=16, YFP-FKBP-ITSN1(DHPH)=28.

Journal of Cell Science • Supplementary information

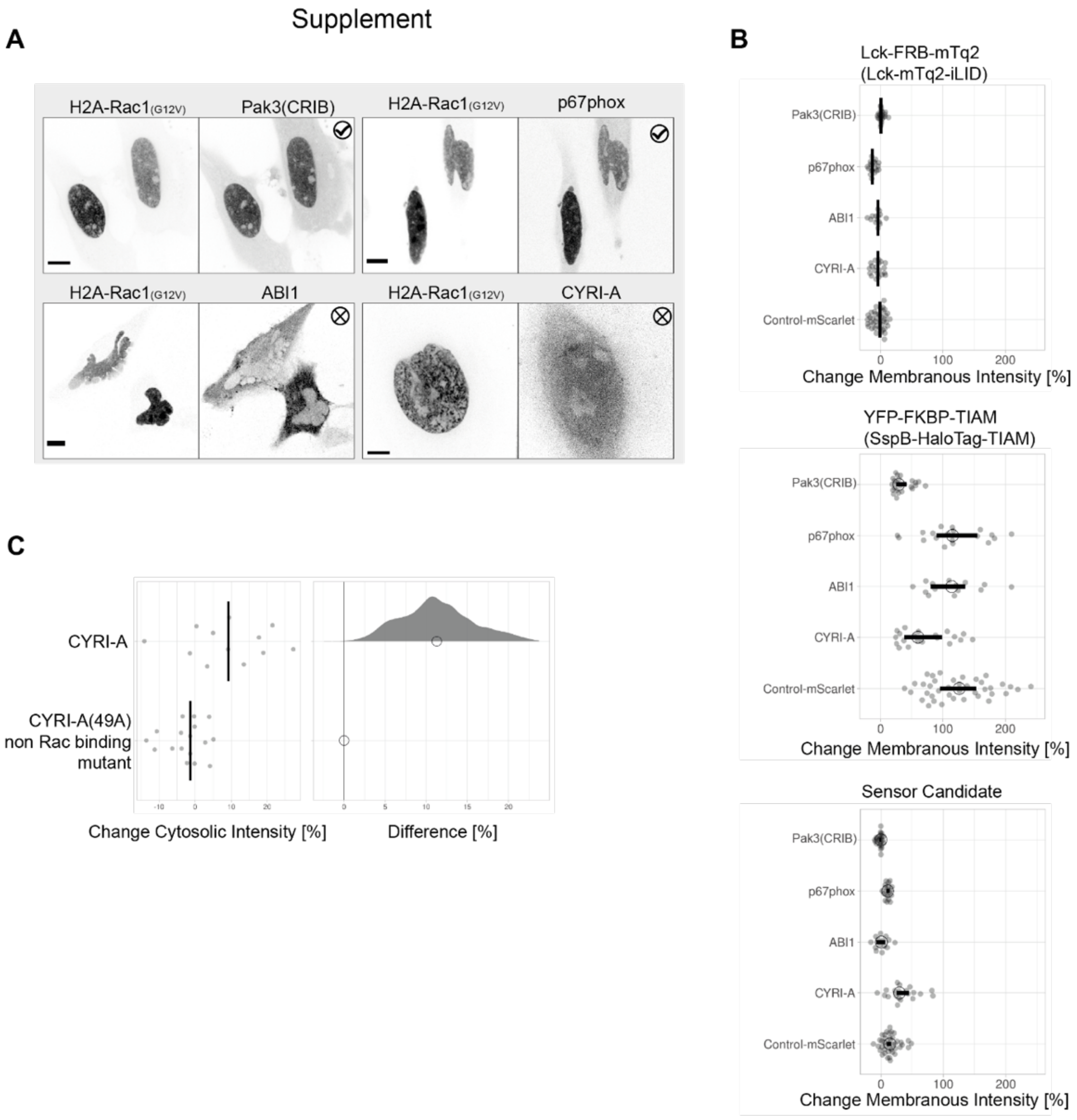

Fig. S4. (A) Spinning disk images of HeLa cells expressing H2A-mTurquoise2-Rac1(G12V)-ΔCaaX (left) and either Rac sensor candidate mCherry-CYRI-A, mScarlet-I-ABI1 or p67phox-mCherry or EGFP-Pak3(CRIB) (right). Check mark indicating colocalization of candidate with nucleus by visual inspection. Scale bars: 10 µm. (B) Change in membranous intensity measured in HeLa cells expressing Lck-FRB-mTurquoise2, YFP-FKBP-TIAM1(DHPH) and either mCherry-CYRI-A, mScarlet-I-ABI1 or p67phox-mCherry, stimulated with 100 nM rapamycin. Additionally, HeLa cells expressing Lck-mTurquoise-iLID, SspB-HaloTag-TIAM1(DHPH) and EGFP-Pak3(CRIB), stimulated with 440 nm laser light at 1% intensity for 1 s in 20 s intervals. Measurements for all three channels, CFP, YFP and RFP. The median is represented as a circle and each dot represents the measurement of a single cell. The 95% confidence interval is represented by a bar. The number of cells measured in two experiments based on independent transfections is: ABI1=14, Control-mScarlet=41, CYRI-A=20, p67phox=19, Pak3(CRIB)=24. The same data is presented in Fig. 6A. (C) Relocation efficiency measured as a change in cytosolic intensity for CYRI-A and the non Rac binding mutant CYRI-A (49A) co-expressed in HeLa cells with Lck-FRB-mTurquoise2 and YFP-FKBP-TIAM1(DHPH) before and after the stimulation with 100 nM rapamycin. For the plot at the left, the median is represented as a black line and each dot

Journal of Cell Science • Supplementary information

represents the measurement of a single cell. The plot at the right shows the effect size relative to the non Rac binding mutant CYRI-A (49A). The bootstrap samples that are used to calculate the 95%CI of the effect size are shown as a distribution. The circle indicates the median. The number of cells measured is: CYRI-A=12, CYRI-A(49A)=17.

Journal of Cell Science • Supplementary information

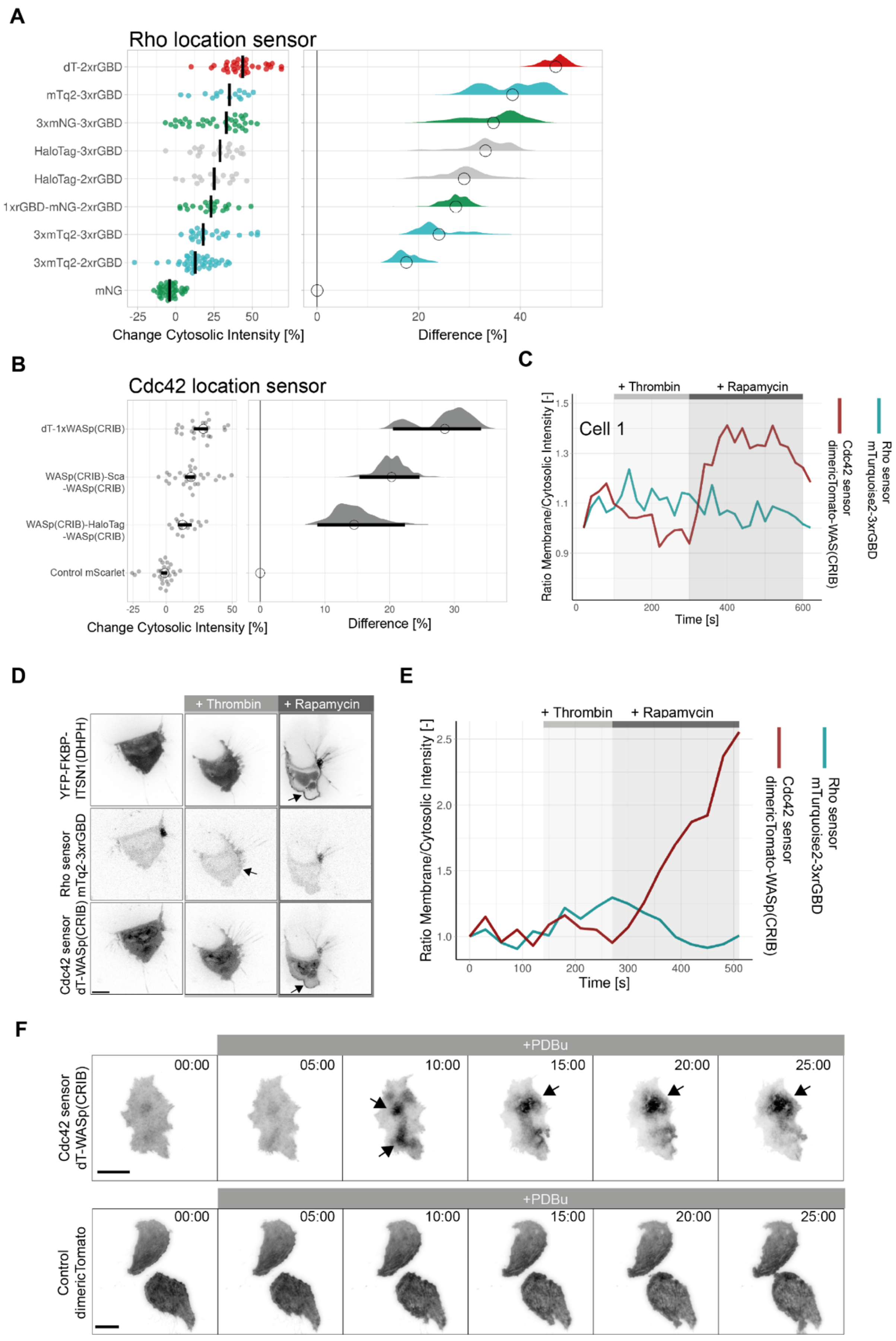

Journal of Cell Science • Supplementary information

Fig. S5. (A) Relocation efficiency plotted as change in cytosolic intensity for CMV-mNeonGreen, CMVdel-3xmTurquoise2-2xrGBD, CMVdel-3xmTurquoise2-3xrGBD, CMVdel-1xrGBD-mNeonGreen-2xrGBD, CMVdel-HaloTag-2xrGBD, CMVdel-HaloTag-3xrGBD, 3xmNeonGreen-3xrGBD, CMVdel-mTurquoise2-3xrGBD and CMVdel-dimericTomato-2xrGBD in HeLa cells co-expressing the histamine 1 receptor (H1R), upon stimulation with 100 µM histamine. For the plot on the left, each dot represents an individual cell and the median of the data is shown as a black bar. The plot at the right shows the effect size relative to the mNeonGreen control. The bootstrap samples that are used to calculate the 95%CI of the effect size are shown as a distribution. The circle indicates the median. The number of cells measured in two experiments based on independent transfections is: 1xrGBD-mNG- 2xrGBD=21, 3xmNG-3xrGBD=34, 3xmTq2-2xrGBD=42, 3xmTq2-3xrGBD=23, dT- 2xrGBD=33, HaloTag-2xrGBD=16, HaloTag-3xrGBD=20, mNG=39, mTq2- 3xrGBD=15. Data for CMV-mNeonGreen and CMVdel-dimericTomato-2xrGBD was taken from (Mahlandt et al., 2021). (B) Relocation efficiency plotted as change in cytosolic intensity for mScarlet-I, WASp(CRIB)-HaloTag-WASp(CRIB), WASp(CRIB)-mScarlet-I-WASp(CRIB) and dimericTomato-WASp(CRIB) in HeLa cells co-expressed with Lck-FRB-mTurquoise2 and YFP-FKBP-ITSN1(DHPH), stimulated with 100 nM rapamycin. For the plot on the left, each dot represents an individual cell and the median of the data is shown as a black bar. The plot at the right shows the effect size relative to the mNeonGreen control. The bootstrap samples that are used to calculate the 95%CI of the effect size are shown as a distribution. The circle indicates the median. The number of cells measured is: mScarlet-I=27, WASp(CRIB)-HaloTag-WASp(CRIB)=15, WASp(CRIB)-mScarlet-I-WASp(CRIB)=27, dimericTomato-WASp(CRIB)=23. Data for mScarlet-I, WASp(CRIB)-mScarlet-I-WASp(CRIB) and dimericTomato-WASp(CRIB) is also shown in Fig. 6C,D,E. (C) Time trace of the ratio membrane over cytosolic intensity for the in Fig. 7B depicted cell 1 for the Rho sensor mTurquoise2-3xrGBD (blue) and the Cdc42 sensor dimericTomato-WASp(CRIB). Thrombin addition is indicated by a light grey bar. Rapamycin addition is indicated by a dark grey bar. D) Spinning disk images of Hek 293T cells expressing Lck-FRB-ECFP(W66A)-black mutant, YFP-FKBP-ITSN1(DHPH) (top), Rho sensor mTurquoise2-3xrGBD (middle) and the Cdc42 sensor dimericTomato-WASp(CRIB) (bottom), first stimulated with 2 U/ml human α-thrombin, and then with 100 nM rapamycin. Arrows indicate intensity increase at the plasma membrane. Scale bar: 10 µm.

Journal of Cell Science • Supplementary information

(E) Time trace of the ratio membrane over cytosolic intensity for the in D depicted cell for the Rho sensor mTurquoise2-3xrGBD (blue) and the Cdc42 sensor dimericTomato-WASp(CRIB). Thrombin addition is indicated by a light grey bar. Rapamycin addition is indicated by a dark grey bar. F) TIRF microscopy images of a SUM159 cell expressing the Cdc42 sensor dimericTomato-WASp(CRIB) (upper panel) or dimericTomato as a control (lower panel), stimulated with PDBu (1 µM), as indicated by the grey bar. Arrows indicate the local signal accumulation of the Cdc42 sensor. Time is given in min:s from the beginning of the recording. Scale bar: 25 µm.

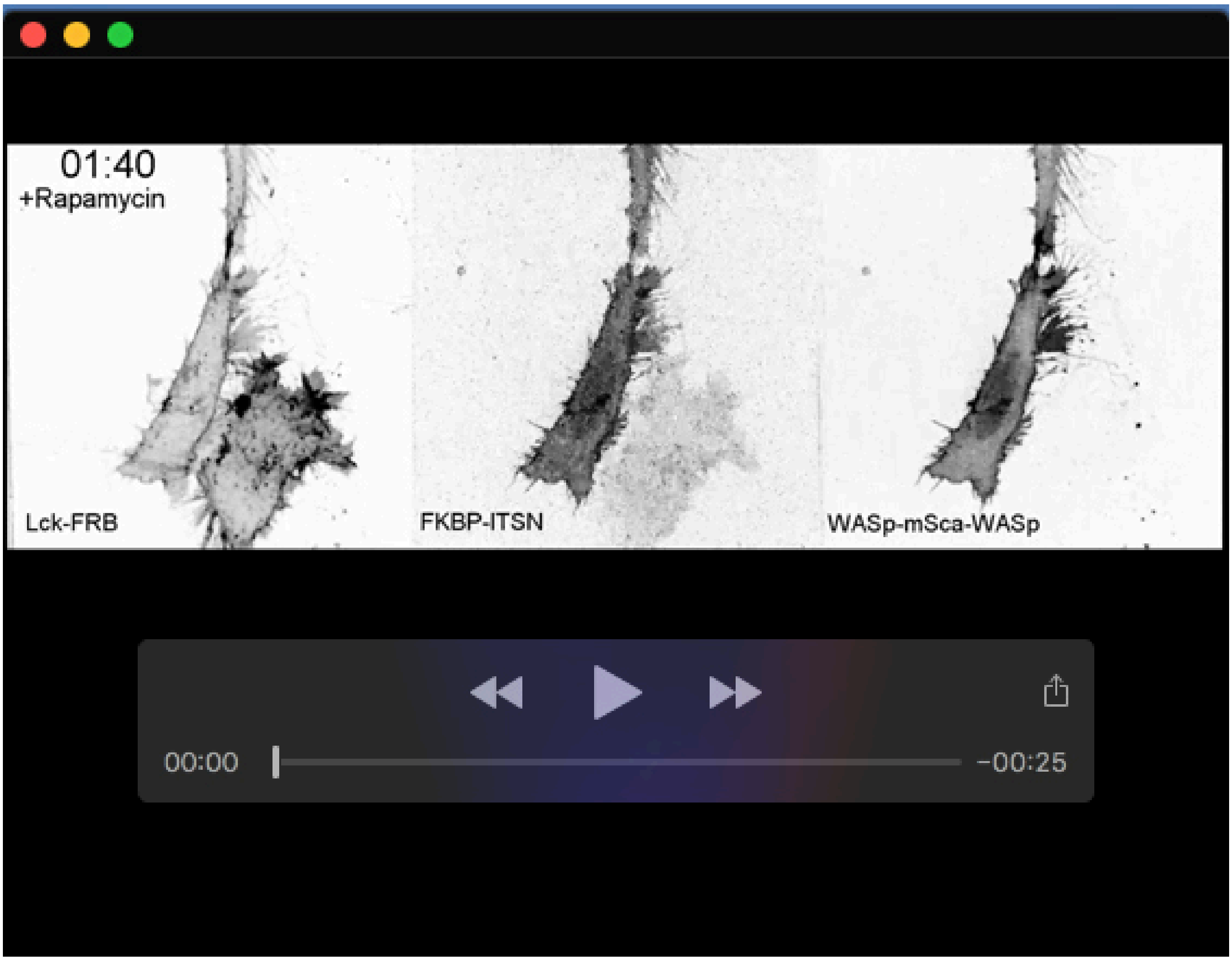

Movie 1. The WASp(CRIB)-mScarlet-I-WASp(CRIB) Cdc42 sensor relocating in a HeLa cell. Spinning disk time lapse movie of a HeLa cell co-expressing Lck-FRB-mTurquoise2 (left), YFP-FKBP-ITSN1(DHPH) (middle) and WASp(CRIB)-mScarlet-I-WASp(CRIB), stimulation with 100 nM rapamycin after 40 s. The time stamper represents min:s from the beginning of the recording.

Journal of Cell Science • Supplementary information

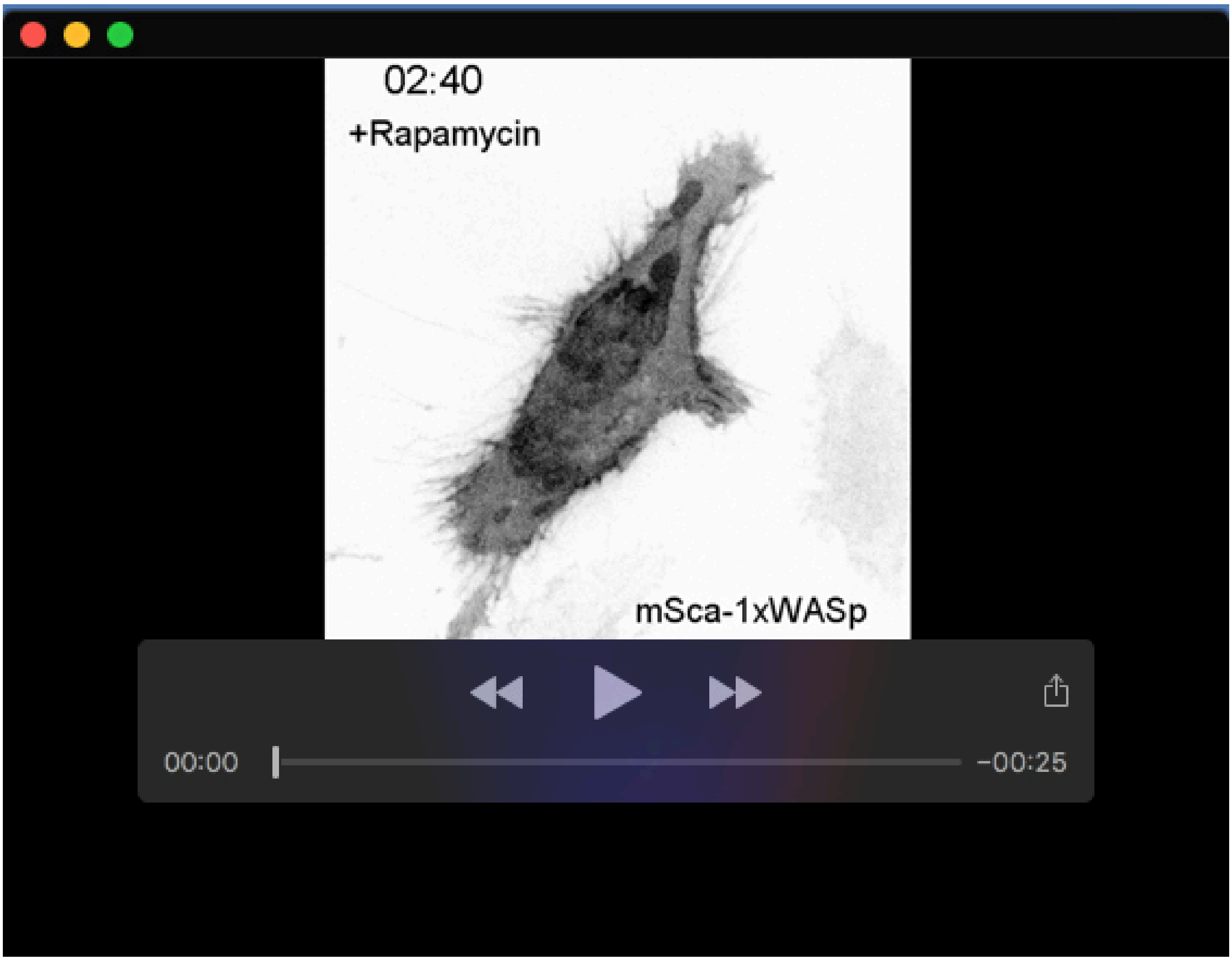

Movie 2. The mScarlet-I-1xWASp(CRIB) Cdc42 sensor relocating in a HeLa cell. Spinning disk microscopy images of a HeLa cell co-expressing Lck-FRB-mTurquoise2 (not shown), YFP-FKBP-ITSN1(DHPH) (not shown) and mScarlet-I-1xWASp(CRIB), stimulated with 100 nM rapamycin at 1:40 min. The time stamper represents min:s from the beginning of the recording.

Journal of Cell Science • Supplementary information

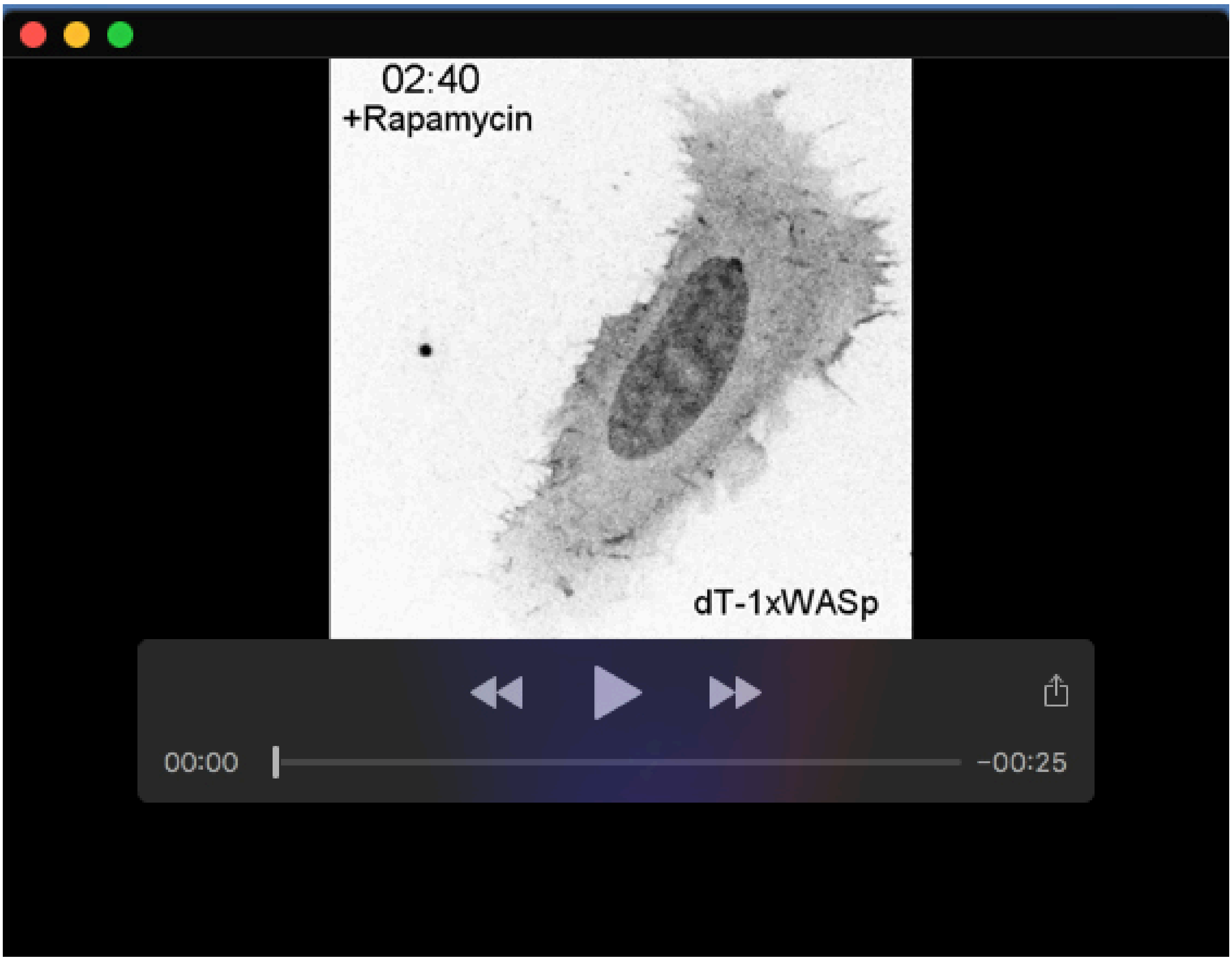

Movie 3. The dimericTomato-1xWASp(CRIB) Cdc42 sensor relocation in a HeLa cell. Spinning disk microscopy images of a HeLa cell (not the cell depicted in Fig. 5B) co-expressing Lck-FRB-mTurquoise2 (not shown), YFP-FKBP-ITSN1(DHPH) (not shown) and dimericTomato-1xWASp(CRIB), stimulated with 100 nM rapamycin at 1:20 min. The time stamper represents min:s from the beginning of the recording.

Journal of Cell Science • Supplementary information

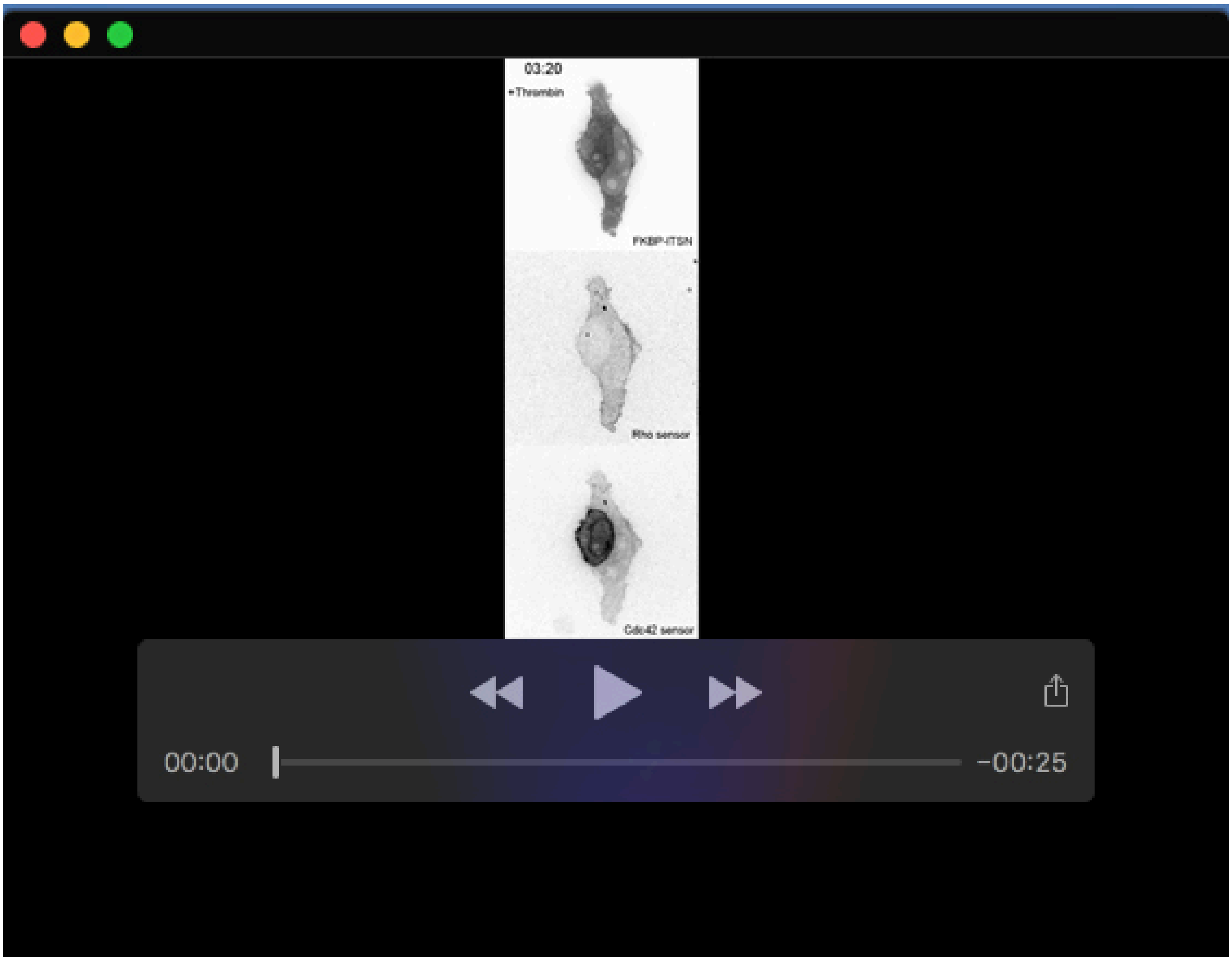

Movie 4. Co-imaging of the optimized Cdc42 and Rho relocation sensors. Spinning disk images of Hek 293T cells co-expressing Lck-FRB-ECFP(W66A)-black mutant (not shown), YFP-FKBP-ITSN1(DHPH) (top), Rho sensor mTurquoise2-3xrGBD (middle) and the Cdc42 sensor dimericTomato-WASp(CRIB) (bottom), first stimulated with 2 U/ml human α-thrombin, and then with 100 nM rapamycin. The time stamper represents min:s from the beginning of the recording.

Journal of Cell Science • Supplementary information

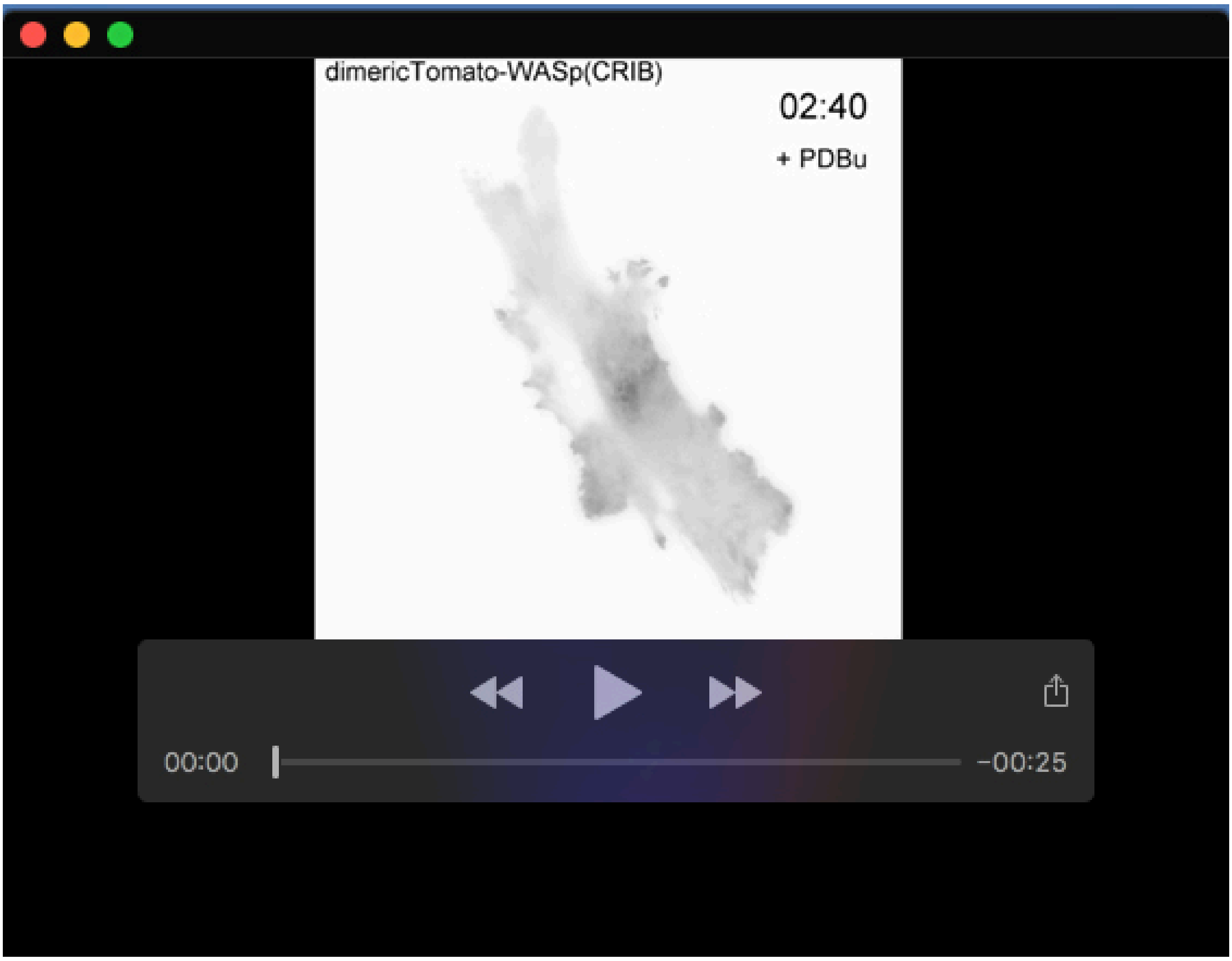

Movie 5. Visualization of local Cdc42 activity. TIRF microscopy time-lapse of a SUM159 cell expressing the Cdc42 sensor dimericTomato-WASp(CRIB) stimulated with PDBu as indicated. Time is given in min:s from the beginning of the recording.

Journal of Cell Science • Supplementary information

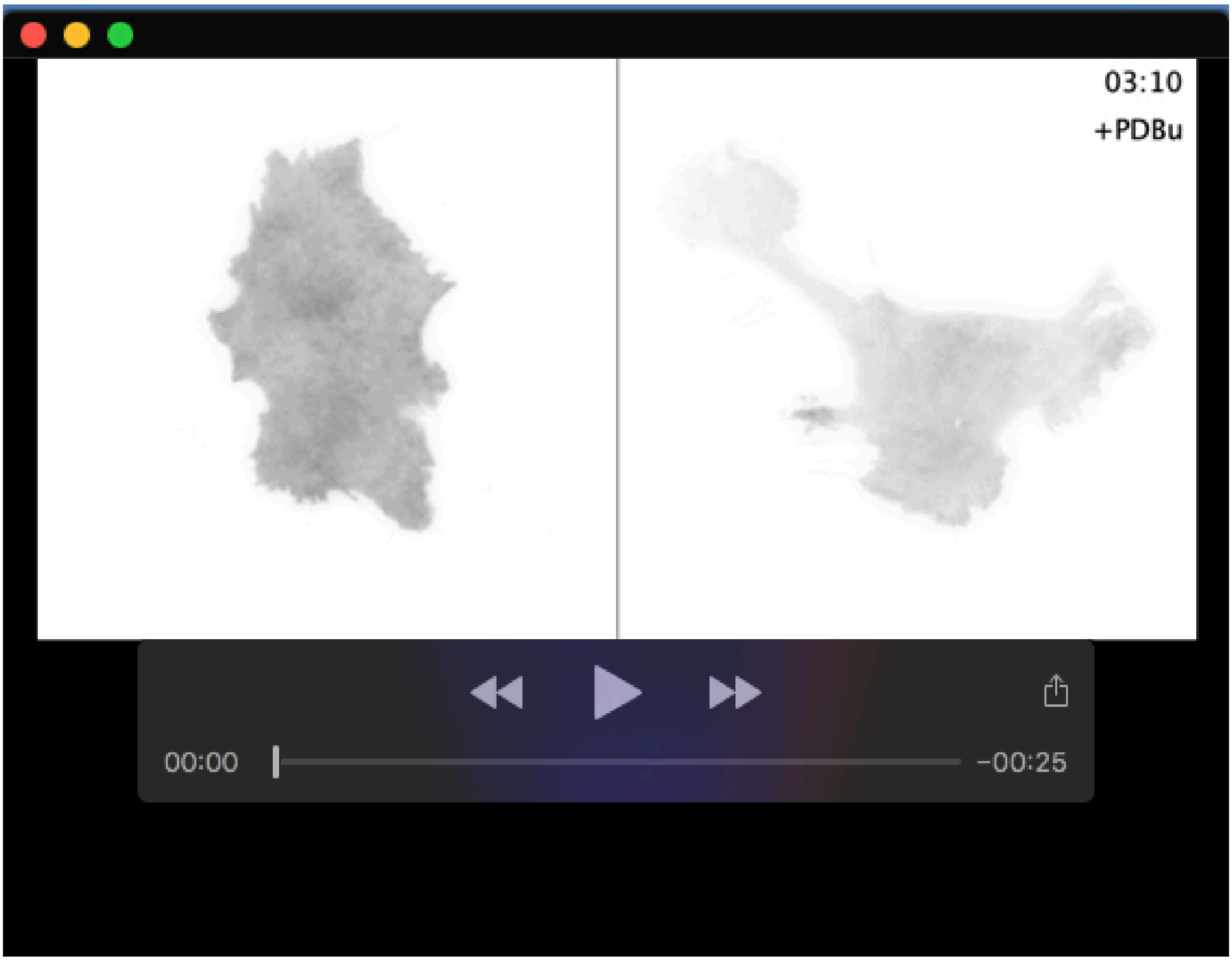

Movie 6. Visualization of local Cdc42 activity. A composite of two TIRF microscopy time-lapses of a SUM159 cell expressing the Cdc42 sensor dimericTomato-WASp(CRIB) stimulated with PDBu as indicated. Time is given in min:s from the beginning of the recording.

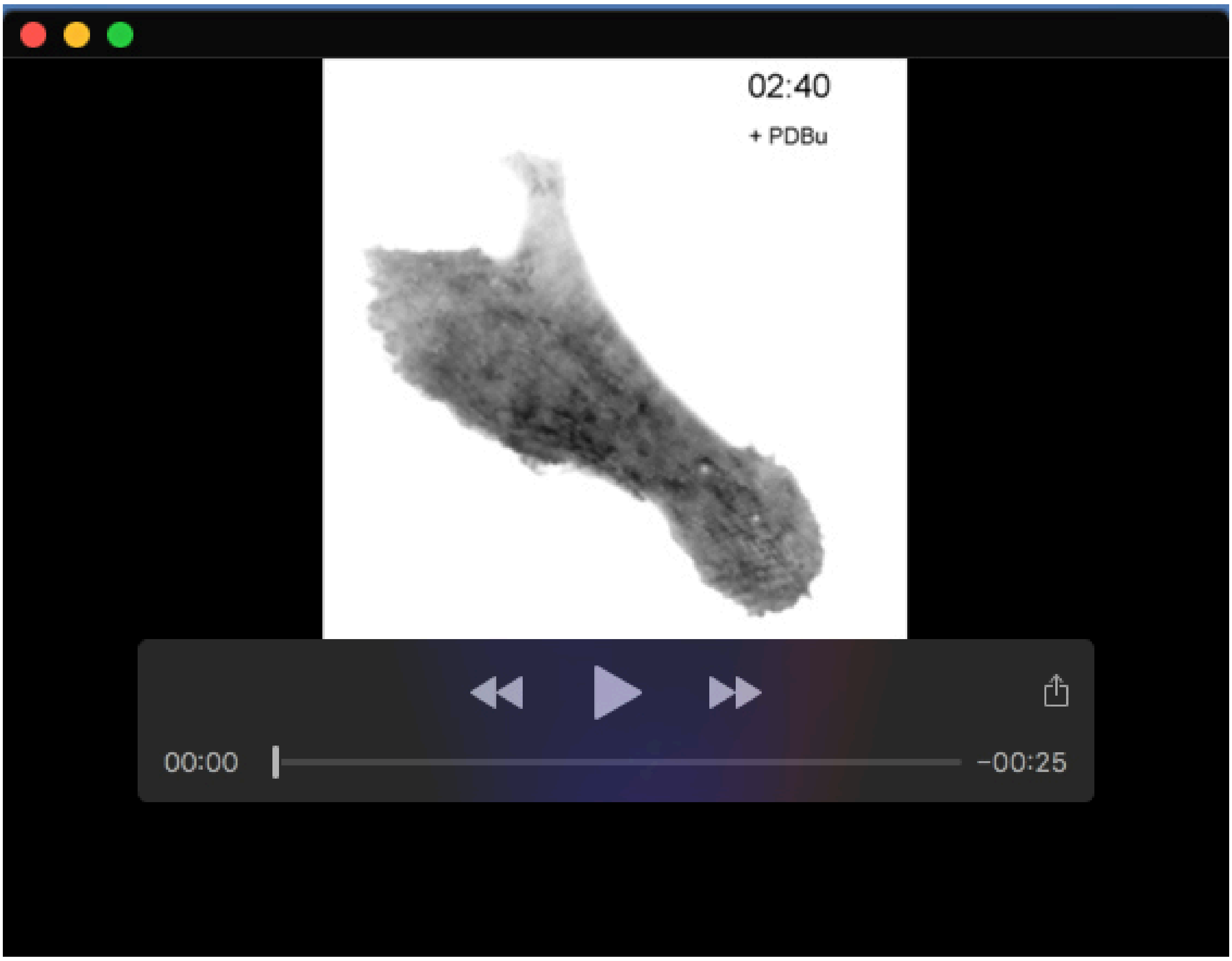

Movie 7. Control for the visualization of local Cdc42 activity. TIRF microscopy time-lapse of a SUM159 cell expressing dimericTomato as a control stimulated with PDBu as indicated. Time is given in min:s from the beginning of the recording.

Journal of Cell Science • Supplementary information
