# Supplementary material for: Cell-based optimization and characterization of genetically encoded location-based biosensors for Cdc42 or Rac activity
Source: J Cell Sci. 2023 May 25;136(10):jcs260802. doi: 10.1242/jcs.260802 (PMC10234108; doi:10.1242/jcs.260802)
Supplement: Supplementary file 1 [file joces-136-260802_review_history.pdf]

Cell-based optimization and characterization of genetically encoded location-based biosensors for Cdc42 or Rac activity

Eike K. Mahlandt, Gabriel Kreider-Letterman, Anna O. Chertkova, Rafael Garcia-Mata and Joachim Goedhart
DOI: 10.1242/jcs.260802

Editor: Michael Way

Review timeline

Submission to Review Commons:	15 September 2022
Submission to Journal of Cell Science:	10 November 2022
Editorial decision:	11 November 2022
First revision received:	12 April 2023
Accepted:	13 April 2023

Reviewer 1

Evidence, reproducibility and clarity

In this manuscript Mahlandt et al. report efforts to generate sensitive fluorescent biosensors to monitor the activation of Rac and Cdc42. While biosensors for these GTPases have been designed and used by others, some have poor specificity while others require the measurement of FRET, which is technically more complex and usually yields low signal to noise ratios. The efforts of Mahlandt et al. are therefore well justified and commendable, and follow on the heels of their successful development of a Rho-selective probe (J. Cell Sci., 2021).

The authors used existing data of interacting proteins to identify candidate domains that could be adapted for use as Rac or Cdc42 activation indicators. They proceeded to select the most promising candidates, assessed their specificity and tried to improve their efficiency by generating tandem constructs that are expected to have higher avidity. They identify CYRA-I as a positive Rac interactor, but the affinity and selectivity of this construct were not deemed to be sufficient and this line of enquiry was not pursued further.
In contrast, the WASp-CRIB was found to be sufficiently Cdc42-specific and its avidity was improved by generating a construct tagged with dimeric Tomato fluorescent protein.
Unfortunately, while data using overexpression of active Cdc42 are convincing and clear, the results obtained under physiological conditions -stimulating cells with S1P- show very modest recruitment. The general usefulness of the probe is therefore questionable.

There are also a number of specific issues:

1. It is not clear why RhoA data were included in this manuscript (Fig. 1), since they seem irrelevant to the primary topic addressed.
2. It is not clear what cell type was used when screening for p67phox. The expression of this component of the NADPH oxidase is restricted to a few specific cell types.
3. There is precious little quantitation of the colocalization or translocation of the probes throughout the manuscript. It is difficult to assess the validity of the conclusions in the absence of analysis of the statistical significance of the colocalization.
4. It is not clear why translocation to mitochondria was used in some experiments and translocation to the nucleus in others.
5. In the S1P experiments, it is difficult to ascertain whether increased fluorescence resulted from membrane folding/ruffling or is actually a consequence of localized activation of receptors. Why

© 2023. Published by The Company of Biologists under the terms of the Creative Commons Attribution License (https://creativecommons.org/licenses/by/4.0/).

does the fluorescence decrease progressively over 1500 seconds? Isn't maximal receptor activation accomplished much sooner?

Significance

While the purpose and intent of the study are commendable, the results are far from convincing and the probes designed do not represent a sufficient improvement over existing biosensors.

The lack of quantitative and statistical analyses is problematic, as is it is difficult to assess the significance of the results.

Reviewer 2

Evidence, reproducibility and clarity

Mahlandt et al report Rho GTPase relocation sensors. First, the authors picked up candidate peptides based on the Mass-Spec data reported by Sean Munro's laboratory. The authors repeated the experiments to confirm the binding of peptides to mitochondria-targeted Cdc42 and Rac1 and narrowed down the candidate peptides by binding to nuclear Cdc42. The specificity of binding to Rac1 and Cdc42 was also tested. Eventually, they concluded that dimeric Tomato-WASp(CRIB) is the best sensor for Cdc42, which could detect S1P-induced Cdc42 activation in primary endothelial cells. The effort to improve the relocation sensors should be evaluated highly. This reviewer has some suggestions to improve this paper.

Major comments:

1. Statistical tests are missing in most of the figures. If the principal purpose of this work is to compare the performance of candidate peptides, the quantitative comparison is essential. If the purpose is just to report another relocation probe, then, more application data may be necessary.

2. The criteria for selecting the best peptide should be clearly described. Is it just by inspection or based on any quantitative data? We know that quantification of colocalization is a difficult task. Therefore, it depends on the aim of this work whether the authors are asked to show quantitative data or not. If a strict comparison of peptides is aimed at, the expression level of each target peptide should be at a comparable level. It will be also required whether the design of each probe guarantees the proper folding to bind to GTPases.

3. About the images of cells: When a fluorescent image is presented, we assume it represents all other cells. Please check all images whether they are truly representing the data. For example, in Fig. S3 the nuclei of ABI1-expressing cells look weird, and the nucleus of CYRI-A is very large. If this is true, the reason why ABI1 and CYRI-A should be excluded from the candidate is not the relocation efficiency but the undesired effect on cell physiology. For the screening of the peptides, this information is also very important. With that, this paper becomes more valuable for scientists.

4. Please examine the order of panels. For example, the result of mScarlet is on the top in Fig3, but at the bottom in Fig4. Such inconsistency would disturb readers.

5. The label should be consistent throughout the paper. For example, in Fig. 5A, Lck-FRB-mTurquoise2 is labeled as Lck-FRB (without the fluorescent protein's name). WASp(CRIB)- mScarlet-I-WASp(CRIB) is labeled as WASp(CRIB)-mScar-WASp(CRIB) (with fluorescent protein's name). Moreover, the same peptide is labeled as mSca-1xWASp(CRIB) in Panel B. Such inconsistency is confusing.

6. Quantitative insight would improve this work. For example, in Fig. 7, the reason why the authors believe that the probe worked is the accumulation of probe at the tip of lamellipodia and the decrease in cytoplasmic intensity. This reviewer does not think the accumulation of the probe in the small area of the lamellipodia explains the massive decrease of cytoplasmic signals. Probably, a substantial amount of the probe is relocated to the plasma membrane, not limited to the lamellipodia.

© 2023. Published by The Company of Biologists under the terms of the Creative Commons Attribution License (https://creativecommons.org/licenses/by/4.0/).

Minor comments:

1. Introduction, "FRET signal is typically measured with a wide field microscope.": This reviewer does not agree with this statement. Confocal and two-photon microscopes have also been used widely.
2. Introduction, "G-protein activating proteins (GAP)": It should read as "GTPase-activating proteins (GAPs)"
3. TRIF should read as TIRF.
4. Fig.1: To the best of this reviewer's knowledge, PKN1 was first used as the RhoA target peptide by Yoshizaki et al in 2003. J Cell Biol 162, 223-232. They also examined mDia, Rhoteki, and Rhophilin as the target peptides. Pak1 was first used as the Rac1 probe by Kraynov et al. Science 290, 333-337, 2000. Use of Pak1 as the Cdc42 probe was reported by Itoh et al. Mol Cell Biol 22, 6582-659, 2002. This reviewer believes that the priority of the first report should be respected.
5. Fig. 1: Why do the authors omit other promising candidates shown in panel 1B? Please describe the reason for the choice.
6. Fig. 1B: Be consistent to use either "Name" or "Uni Prot name" in Panel A.
7. Fig. 2: Please include information on TOMM20. The readers may not read the paper by Gillingham et al.
8. Fig3 and 4: The authors should show the images of control H2A.
9. In Fig3B and 4B, "Cdc42/Rac1 affinity" would be misleading, because the control dots represent their authentic localization rather than "Cdc42/Rac1 affinity".
10. Fig. 4: More explanation of this figure is required.
11. Fig. 5: More explanation about the FKBP-FRB system will be helpful.
12. Fig. 6: It is rather surprising to see that control-mScarlet also responds to Rac1 activation. What is the explanation for this observation?
13. Fig. 7: A single champion data may not be convincing to prove the usefulness of this probe

Significance

1. The authors have screened many peptides, which may serve as the relocation sensor for Rho-family GTPases.
2. There are precedent relocation sensors, a part of which is listed in Fig. 1A. This work discloses an improved relocation biosensor.
3. Cell biologists who is working on Cdc42 will be interested in this probe.
4. Expertise of this reviewer: Signal transduction, Fluorescence microscopy.

Reviewer 3

Evidence, reproducibility and clarity

The manuscript was clearly written, but the introduction or discussion could provide a more balanced view of the significance. There are important experiments that are required to support the conclusions regarding selectivity and differences between the domains, particularly the role of expression level and the ratio of expressed proteins. Our review is summarized here:

The authors set out to improve localization-based biosensors for Rac1 and Cdc42 by identifying domains that bind selectively to the activated conformation of Rac1 and Cdc42. Using screens based on mitochondrial and nuclear relocalization, together with mass spec and proximity biotinylation, several potential candidates were identified. The work concludes with the identification of a WASp CRIB domain that can be used as a useful Cdc42 relocalization biosensor. It was applied in a well-executed proof of principle study demonstrating multiplexed imaging of RhoA and Cdc42 localization biosensors.

1. The discussion comparing different types of biosensors missed important points. Although the advantages of localization biosensors listed by the authors are correct, they gave the impression that these should simply be an improved replacement for FRET biosensors. There are times when FRET biosensors provide clear advantages. Unlike other proteins, Rho GTPases are well suited for localization sensors because the activated conformation, and only the activated conformation,

© 2023. Published by The Company of Biologists under the terms of the Creative Commons Attribution License (https://creativecommons.org/licenses/by/4.0/).

localizes to the membrane. For diffuse or 3D localization FRET can provide better quantification. Subtle features such as gradients are not easily quantified over a background of unattached domain. The authors state that localization biosensors have enhanced spatial resolution, but this is not explained.

2. Throughout the paper, the ratio between the GTPase and the domain, and the overall expression level of each, was not sufficiently examined. The results in many cases would be dependent on both these factors (was a large excess of domain used? Was there insufficient domain to bind the GTPase and provide a signal? Did this vary for different domains, and therefore produce the differences observed? A lack of apparent binding specificity could be produced by high domain expression.)

3. In the nuclear exclusion assay, some GTPases were excluded from the nucleus and others not. This was true even without expression of the domains. When GTPases were excluded from the nucleus, domains were eliminated from contention, even when this was true without domain. The authors could at least mention that these domains may be viable.

4. In the multiplexing experiment, only two cells were imaged. In one cell RhoA activity was inversely correlated with Cdc42 activity. In the other cell it was not. It seems there is insufficient information to reach firm conclusions.

Minor points:
- There appear to be errors in naming mutants. Q60L is used for constitutively active Rac, but Q61L is likely meant. H2A-mTurquoise2-Rac1(G12V)-?CaaX is used when it likely should be H2A-mTurquoise2-Rac1(Q61L)-?CaaX. There are other examples -- a careful check of these names throughout the manuscript would be valuable.
- Intro-Paragraph 1-line 5: change present to presence
- Intro-Paragraph 5- line 7: use them instead of theme.

Significance

The assays and approach to finding domains for biosensors were novel and interesting. The end result was not as surprising as one might have hoped, but the approach alone made the paper worthwhile.

Author response to reviewers' comments

1. General Statements [optional]
We were pleased with the overall positive comments of the reviewers:
-Reviewer #1: "The efforts of Mahlandt et al. are therefore well justified and commendable"
-Reviewer #2: "The effort to improve the relocation sensors should be evaluated highly"
-Reviewer #3: "The assays and approach to finding domains for biosensors were novel and interesting. The end result was not as surprising as one might have hoped, but the approach alone made the paper worthwhile."

We thank the reviewers for their fair and constructive comments that will help us to improve the manuscript.

2. Description of the planned revisions

We propose three revisions, that have not yet been included in the current manuscript:

1. All three reviewers comment on the data in figure 7, in which the application of the sensor is shown. We agree that the number of cells is low, and we plan to repeat this experiment to increase the number of cells, and better demonstrate the usefulness of the new probe. We note that the improved Cdc42 sensor is used in a recent preprint (see figures 7 and 9 of: https://www.biorxiv.org/content/10.1101/2022.06.22.497207v2.full), clearly showing the potential of the probe for detection of Cdc42 and increasing our confidence that we can generate

© 2023. Published by The Company of Biologists under the terms of the Creative Commons Attribution License (https://creativecommons.org/licenses/by/4.0/).

higher quality data.

2. The ratio of expression of the different components was not quantified. We have these data and we will (re)analyze it and present the results (related to Reviewer #3, point2).

3. We will reanalyze the images to ensure that representative images are depicted in the manuscript (related to Reviewer #2, point 3).

Next to new experiments/analyses mentioned here, we will address all comments and incorporate all the suggestions by the reviewers.

3. Description of the revisions that have already been incorporated in the transferred manuscript

Reviewer #1

1) It is not clear why RhoA data were included in this manuscript (Fig. 1), since they seem irrelevant to the primary topic addressed.

We have cell-based data from our previous (published) work that we can use to check whether these results align with the mass-spec data. To make this point clearer we add "We looked first into GBDs for Rho, to compare the results of the mass spectrometry screen with the results of our cell-based assays".

2) It is not clear what cell type was used when screening for p67phox. The expression of this component of the NADPH oxidase is restricted to a few specific cell types.

That's a relevant point and therefore observation that p67phox is not detected is perhaps not surprising. We removed this statement.

3) There is precious little quantitation of the colocalization or translocation of the probes throughout the manuscript. It is difficult to assess the validity of the conclusions in the absence of analysis of the statistical significance of the colocalization.

In figure 2, which is an initial screen, there is only a qualitative assessment. However, for the promising candidates, there is a quantitative assessment in Figures 3B and 4 B as to which extent the candidates colocalize with the nuclear localized target. From the rank order and individual datapoints the best performing binder can be inferred.

4) It is not clear why translocation to mitochondria was used in some experiments and translocation to the nucleus in others.

To clarify, we have added text: "We have previously used nuclear localized, constitutive active Rho GTPases, but these are not accessible for larger proteins that cannot enter the nucleus"

5) In the S1P experiments, it is difficult to ascertain whether increased fluorescence resulted from membrane folding/ruffling or is actually a consequence of localized activation of receptors. Why does the fluorescence decrease progressively over 1500 seconds? Isn't maximal receptor activation accomplished much sooner?

This experiment suffered from bleaching. We will redo the experiment to get higher number of cells and to improve the data.

Reviewer #2

1. Statistical tests are missing in most of the figures. If the principal purpose of this work is to compare the performance of candidate peptides, the quantitative comparison is essential. If the purpose is just to report another relocation probe, then, more application data may be necessary.

© 2023. Published by The Company of Biologists under the terms of the Creative Commons Attribution License (https://creativecommons.org/licenses/by/4.0/).

We will improve the quality of the application data. As for statistics, we have added the effect size to figures 5C-F and figure 6A. To explain this (not so common) statistic we add to the materials and methods: "The effect size that quantifies the difference and its distribution was calculated with the web tool 'PlotsofDifferences'".

2. The criteria for selecting the best peptide should be clearly described. Is it just by inspection or based on any quantitative data? We know that quantification of colocalization is a difficult task. Therefore, it depends on the aim of this work whether the authors are asked to show quantitative data or not. If a strict comparison of peptides is aimed at, the expression level of each target peptide should be at a comparable level. It will be also required whether the design of each probe guarantees the proper folding to bind to GTPases.

There are two stages for the selection. First, we did a qualitative analysis of colocalization (shown in figure 2). Based on the results ("Candidates colocalizing with the mitochondrial tagged Rho GTPase were further tested for their potential as localization-based sensors"), we generated smaller biosensor candidates of which binding to a nuclear target was quantitatively analyze (figures 3B and 4B). As the expression level is an important factor, we ascertained potential candidates were expressed at roughly the same level in the nuclear accumulation assay.

3. About the images of cells: When a fluorescent image is presented, we assume it represents all other cells. Please check all images whether they are truly representing the data. For example, in Fig. S3 the nuclei of ABI1-expressing cells look weird, and the nucleus of CYRI-A is very large. If this is true, the reason why ABI1 and CYRI-A should be excluded from the candidate is not the relocation efficiency but the undesired effect on cell physiology. For the screening of the peptides, this information is also very important. With that, this paper becomes more valuable for scientists.

We agree that this is an important point. We will reanalyze the data as indicated in the 'planned revisions'.

4. Please examine the order of panels. For example, the result of mScarlet is on the top in Fig3, but at the bottom in Fig4. Such inconsistency would disturb readers.

We thank the reviewer for this suggestion and we changed figure 4.

5. The label should be consistent throughout the paper. For example, in Fig. 5A, Lck-FRB-mTurquoise2 is labeled as Lck-FRB (without the fluorescent protein's name). WASp(CRIB)-mScarlet-I-WASp(CRIB) is labeled as WASp(CRIB)-mScar-WASp(CRIB) (with fluorescent protein's name). Moreover, the same peptide is labeled as mSca-1xWASp(CRIB) in Panel B. Such inconsistency is confusing.

We agree, we have updated figure 5A by adding the abbreviations of the fluorescent proteins. Please note that WASp(CRIB)-mSca-WASp(CRIB), mSca-1xWASp(CRIB) and mSca- 2xWASp(CRIB) are three different constructs. In the first one the CRIB domains are sandwiching the fluorescent protein and in the third one they are in tandem downstream of the fluorescent protein.

6. Quantitative insight would improve this work. For example, in Fig. 7, the reason why the authors believe that the probe worked is the accumulation of probe at the tip of lamellipodia and the decrease in cytoplasmic intensity. This reviewer does not think the accumulation of the probe in the small area of the lamellipodia explains the massive decrease of cytoplasmic signals. Probably, a substantial amount of the probe is relocated to the plasma membrane, not limited to the lamellipodia.

We propose to repeat the experiment shown in figure 7 and to improve the quality of the data.

1. Introduction, "FRET signal is typically measured with a wide field microscope.": This reviewer does not agree with this statement. Confocal and two-photon microscopes have also been used widely.

© 2023. Published by The Company of Biologists under the terms of the Creative Commons Attribution License (https://creativecommons.org/licenses/by/4.0/).

Fair point. We changed the text to "when the FRET signal is measured with a wide field microscope"

2. Introduction, "G-protein activating proteins (GAP)": It should read as "GTPase-activating proteins (GAPs)"

Thanks, corrected.

3. TRIF should read as TIRF.

All instances have been corrected.

4. Fig.1: To the best of this reviewer's knowledge, PKN1 was first used as the RhoA target peptide by Yoshizaki et al in 2003. J Cell Biol 162, 223-232. They also examined mDia, Rhoteki, and Rhophilin as the target peptides. Pak1 was first used as the Rac1 probe by Kraynov et al. Science 290, 333-337, 2000. Use of Pak1 as the Cdc42 probe was reported by Itoh et al. Mol Cell Biol 22, 6582-659, 2002. This reviewer believes that the priority of the first report should be respected.

We changed part of the introduction to:
High scoring proteins for interacting with constitutively active RhoA(Q63L) included ANLN part of the AniRBD Rho location sensor (Piekny and Glotzer, 2000), PKN1 part of aRho FRET sensor (Yoshizaki et al., 2003) and RTKN part of the rGBD Rho location sensor (Benink and Bement, 2005; Mahlandt et al., 2021) (Fig. 1A,B). This suggested that proteins with a high score in the mass spectrometry screen are potentially suitable as Rho GTPase activity biosensor. Indeed, the GBDs used for Cdc42 location sensors from, PAK1 used in the PBD location sensor (Itoh et al., 2002; Petrie et al., 2012) and N-WASP similar to WASp used in the wGBD location sensor (Benink and Bement, 2005) showed a high score in the screen (Fig. 1A,B).

Discussion:
Another challenge is the Rho GTPase specificity of the relocation-based sensor. For example, Pak1(CRIB) was first used in a Rac1 FRET sensor (Kraynov et al., 2000). ThenPak1(CRIB) has been utilized in Cdc42 FRET sensors and in an intensiometric Cdc42 sensor (Hanna et al., 2014; Itoh et al., 2002; Kim et al., 2019). However, Pak1(CRIB), also named PBD sensor, has then been reintroduced by Weiner and colleagues as a Rac1 specific location-based sensor and is often used in neutrophil HL60 cells (Brunetti et al., 2022; Graziano et al., 2019; Le et al., 2021; Weiner et al., 2007).

We also updated the tables in Figure 1.

5. Fig. 1: Why do the authors omit other promising candidates shown in panel 1B? Please describe the reason for the choice.

We took into account the availability of plasmid DNA, as also explained in the manuscript: "candidate GBDs were selected from top 30 scores of the mass spectrometry screen, that were specific for one Rho GTPase and their DNA was available on addgene"

6. Fig. 1B: Be consistent to use either "Name" or "Uni Prot name" in Panel A.

We updated figure 1.

7. Fig. 2: Please include information on TOMM20. The readers may not read the paper by Gillingham et al.

We added an explanation: "To this end, a fusion with TOMM20 was used for mitochondrial localization."

8. Fig3 and 4: The authors should show the images of control H2A.

We provide the data for control H2A in figures 3B and 4B.

© 2023. Published by The Company of Biologists under the terms of the Creative Commons Attribution License (https://creativecommons.org/licenses/by/4.0/).

9. In Fig3B and 4B, "Cdc42/Rac1 affinity" would be misleading, because the control dots represent their authentic localization rather than "Cdc42/Rac1 affinity".

We agree, we have updated figure 3B and 4B.

10. Fig. 4: More explanation of this figure is required.

We added text: "Hence, the sensor candidate can freely partition between Rac and Cdc42 binding."

11. Fig. 5: More explanation about the FKBP-FRB system will be helpful.

We changed the text to: "The system used rapamycin induced heterodimerization of the two domains FRB and FKBP to recruit the DHPH domain of the Cdc42 specific GEF ITSN1 to the plasma membrane, where it induces activity of the endogenous Cdc42"

12. Fig. 6: It is rather surprising to see that control-mScarlet also responds to Rac1 activation. What is the explanation for this observation?

We agree and have no explanation.

13. Fig. 7: A single champion data may not be convincing to prove the usefulness of this probe.

We agree and propose to repeat the experiment.

Reviewer #3

1) The discussion comparing different types of biosensors missed important points. Although the advantages of localization biosensors listed by the authors are correct, they gave the impression that these should simply be an improved replacement for FRET biosensors. There are times when FRET biosensors provide clear advantages. Unlike other proteins, Rho GTPases are well suited for localization sensors because the activated conformation, and only the activated conformation, localizes to the membrane. For diffuse or 3D localization FRET can provide better quantification. Subtle features such as gradients are not easily quantified over a background of unattached domain. The authors state that localization biosensors have enhanced spatial resolution, but this is not explained.

We agree that our introduction is biased towards a preference for relocation based biosensors. However, having used both approaches, we see that both strategies have pro's and cons. Therefore, we removed the claim for higher resolution and we added: "Still, the ratiometric mode of imaging FRET sensors is beneficial for detection of gradients or activity in 3D imaging".

2) Throughout the paper, the ratio between the GTPase and the domain, and the overall expression level of each, was not sufficiently examined. The results in many cases would be dependent on both these factors (was a large excess of domain used? Was there insufficient domain to bind the GTPase and provide a signal? Did this vary for different domains, and therefore produce the differences observed? A lack of apparent binding specificity could be produced by high domain expression.)

This is an important point. We will re-analyze the data and include a figure where we add the binding efficiency versus the expression level.

3) In the nuclear exclusion assay, some GTPases were excluded from the nucleus and others not. This was true even without expression of the domains. When GTPases were excluded from the nucleus, domains were eliminated from contention, even when this was true without domain. The authors could at least mention that these domains may be viable.

Correct, and we have added this text: "we cannot exclude that these would be viable Cdc42 sensor candidates"

© 2023. Published by The Company of Biologists under the terms of the Creative Commons Attribution License (https://creativecommons.org/licenses/by/4.0/).

4) In the multiplexing experiment, only two cells were imaged. In one cell RhoA activity was inversely correlated with Cdc42 activity. In the other cell it was not. It seems there is insufficient information to reach firm conclusions.

We agree and in the revision plan we indicate that we will repeat this experiment to increase the number of cells.

Minor points:

- There appear to be errors in naming mutants. Q60L is used for constitutively active Rac, but Q61L is likely meant. H2A-mTurquoise2-Rac1(G12V)-?CaaX is used when it likely should be H2A-mTurquoise2-Rac1(Q61L)-?CaaX. There are other examples -- a careful check of these names throughout the manuscript would be valuable.

Thanks for spotting this. Q60L is changed to Q61L. Note that the Rac1(G12V) is correct as it also is a constitutive active Rac1.

- Intro-Paragraph 1-line 5: change present to presence
- Intro-Paragraph 5- line 7: use them instead of theme.

Thanks, both corrected.

4. Description of analyses that authors prefer not to carry out

None

Original submission

First decision letter

MS ID#: JOCES/2022/260802

MS TITLE: A systematic, cell-based comparison of G-protein binding domains for their potential as localization-based, genetically encoded biosensors of Cdc42 or Rac activity

AUTHORS: Eike K. Mahlandt, Anna O. Chertkova, and Joachim Goedhart

ARTICLE TYPE: Research Article

We have now reached a decision on the above review commons manuscript.

To see the reviewers' reports and a copy of this decision letter, please go to: https://submit-jcs.biologists.org and click on the 'Manuscripts with Decisions' queue in the Author Area. (Corresponding author only has access to reviews.)

As you will have seen, the reviewers raise a number of substantial criticisms. Your response to the comments is appropriate and I would be pleased to see a revised manuscript that contains the correction and data you have indicated in your response to the reviewers concerns.
Please ensure that you clearly highlight all changes made in the revised manuscript. Please avoid using 'Tracked changes' in Word files as these are lost in PDF conversion.

I should be grateful if you would also provide a point-by-point response detailing how you have dealt with the points raised by the reviewers in the 'Response to Reviewers' box. Please attend to all of the reviewers' comments. If you do not agree with any of their criticisms or suggestions please explain clearly why this is so.

© 2023. Published by The Company of Biologists under the terms of the Creative Commons Attribution License (https://creativecommons.org/licenses/by/4.0/).

First revision

Author response to reviewers' comments

We thank the reviewers for their fair and constructive comments that have helped us to substantially improve manuscript. The main concern was related to the application of the new biosensor (as shown in figure 7), which showed only a single cell. We have added new data, obtained by a collaboration with Gabriel Kreider-Letterman and Rafael Garcia-Mata (University of Toledo, Toledo, OH, USA), who we have added as co-authors. The new data of multiple cells (including new supplementary movies 5,6,7) nicely demonstrate that application of the probe for detection of Cdc42 activity, with good spatiotemporal resolution and high contrast. We now also include a control, untagged sensor for this data. We think that the new data this is a valuable and convincing addition to the manuscript.

Below we reply to all comments, point-by-point:

Reviewer #1

1) It is not clear why RhoA data were included in this manuscript (Fig. 1), since they seem irrelevant to the primary topic addressed.

We have cell-based data from our previous (published) work that we can use to check whether these results align with the mass-spec data. To make this point clearer we add "We looked first into GBDs for Rho, to compare the results of the mass spectrometry screen with the results of our cell-based assays".

2) It is not clear what cell type was used when screening for p67phox. The expression of this component of the NADPH oxidase is restricted to a few specific cell types.

That's a relevant point and therefore observation that p67phox is not detected is perhaps not surprising. Although this probe has successfully been used in U2OS cells.

3) There is precious little quantitation of the colocalization or translocation of the probes throughout the manuscript. It is difficult to assess the validity of the conclusions in the absence of analysis of the statistical significance of the colocalization.

In figure 2, which is an initial screen, there is only a qualitative assessment. However, for the promising candidates, there is a quantitative assessment in Figures 3B and 4 B as to which extent the candidates colocalize with the nuclear localized target. From the rank order and individual datapoints the best performing binder can be inferred.

4) It is not clear why translocation to mitochondria was used in some experiments and translocation to the nucleus in others.

To clarify, we have added text: "We have previously used nuclear localized, constitutive active Rho GTPases, but these are not accessible for larger proteins that cannot enter the nucleus"

5) In the S1P experiments, it is difficult to ascertain whether increased fluorescence resulted from membrane folding/ruffling or is actually a consequence of localized activation of receptors. Why does the fluorescence decrease progressively over 1500 seconds? Isn't maximal receptor activation accomplished much sooner?

This experiment suffered from bleaching. We changed to the observation of Cdc42 activity at invadopodia with TIRF microscopy, which is less influenced by intensity changes caused by membrane ruffling.

© 2023. Published by The Company of Biologists under the terms of the Creative Commons Attribution License (https://creativecommons.org/licenses/by/4.0/).

Reviewer #2

1. Statistical tests are missing in most of the figures. If the principal purpose of this work is to compare the performance of candidate peptides, the quantitative comparison is essential. If the purpose is just to report another relocation probe, then, more application data may be necessary.

We have added more application data and included a control. As for statistics, we have added the effect size to figures 5C-F and figure 6A. To explain this (not so common) statistic we add to the materials and methods: "The effect size that quantifies the difference and its distribution was calculated with the web tool 'PlotsofDifferences'".

2. The criteria for selecting the best peptide should be clearly described. Is it just by inspection or based on any quantitative data? We know that quantification of colocalization is a difficult task. Therefore, it depends on the aim of this work whether the authors are asked to show quantitative data or not. If a strict comparison of peptides is aimed at, the expression level of each target peptide should be at a comparable level. It will be also required whether the design of each probe guarantees the proper folding to bind to GTPases.

There are two stages for the selection. First, we did a qualitative analysis of colocalization (shown in figure 2). Based on the results ("Candidates colocalizing with the mitochondrial tagged Rho GTPase were further tested for their potential as localization-based sensors"), we generated smaller biosensor candidates of which binding to a nuclear target was quantitatively analyze (figures 3B and 4B). As the expression level is an important factor, we ascertained potential candidates were expressed at roughly the same level in the nuclear accumulation assay.

3. About the images of cells: When a fluorescent image is presented, we assume it represents all other cells. Please check all images whether they are truly representing the data. For example, in Fig. S3 the nuclei of ABI1-expressing cells look weird, and the nucleus of CYRI-A is very large. If this is true, the reason why ABI1 and CYRI-A should be excluded from the candidate is not the relocation efficiency but the undesired effect on cell physiology. For the screening of the peptides, this information is also very important. With that, this paper becomes more valuable for scientists.

We agree that this is an important point.

Concerning ABI1, it was not uncommon to see these 'constrictions' in the nucleus. They can also be seen in Fig. 3C for Bem3(CRIB2), CDC42EPI(CRIB2) and Pak4(CRIB). However, they seem more pronounced in the ABI1 images. Here is another example, where the nucleus looks more even.

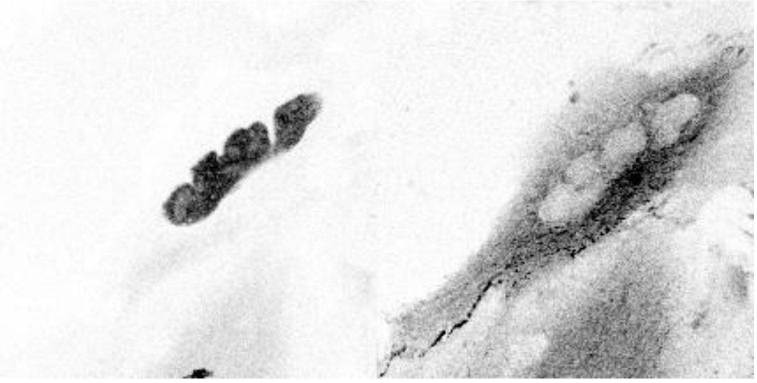

(Left: H2A-constitutivley active Rac1, Right: ABI1)

© 2023. Published by The Company of Biologists under the terms of the Creative Commons Attribution License (https://creativecommons.org/licenses/by/4.0/).

Concerning CYR, this construct seems to indeed enlarge the nucleus and alter its morphology.

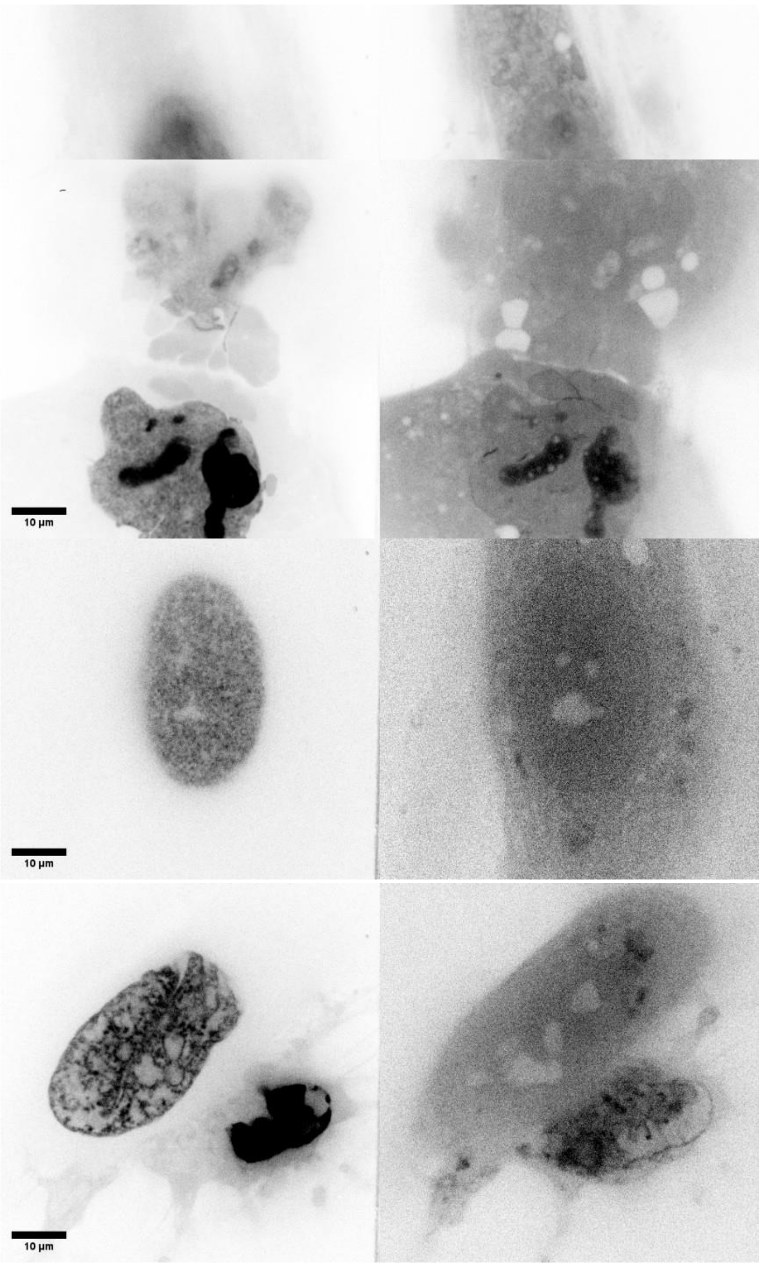

(Left: H2A-constitutivley active Rac1, Right: CYRI-A)

We added to the text: 'Additionally, CYRI-A seemed to change the morphology of the nucleus and increase its size.'

4. Please examine the order of panels. For example, the result of mScarlet is on the top in Fig3, but at the bottom in Fig4. Such inconsistency would disturb readers.

We thank the reviewer for this suggestion and we changed figure 4.

5. The label should be consistent throughout the paper. For example, in Fig. 5A, Lck-FRB-mTurquoise2 is labeled as Lck-FRB (without the fluorescent protein's name). WASp(CRIB)- mScarlet-I-WASp(CRIB) is labeled as WASp(CRIB)-mScar-WASp(CRIB) (with fluorescent protein's name). Moreover, the same peptide is labeled as mSca-1xWASp(CRIB) in Panel B. Such inconsistency is confusing.

© 2023. Published by The Company of Biologists under the terms of the Creative Commons Attribution License (https://creativecommons.org/licenses/by/4.0/).

We agree, we have updated figure 5A by adding the abbreviations of the fluorescent proteins. Please note that WASp(CRIB)-mSca-WASp(CRIB), mSca-1xWASp(CRIB) and mSca- 2xWASp(CRIB) are three different constructs. In the first one the CRIB domains are sandwiching the fluorescent protein and in the third one they are in tandem downstream of the fluorescent protein.

6. Quantitative insight would improve this work. For example, in Fig. 7, the reason why the authors believe that the probe worked is the accumulation of probe at the tip of lamellipodia and the decrease in cytoplasmic intensity. This reviewer does not think the accumulation of the probe in the small area of the lamellipodia explains the massive decrease of cytoplasmic signals. Probably, a substantial amount of the probe is relocated to the plasma membrane, not limited to the lamellipodia.

We agree that this data was not very convincing. So we have added new data that more clearly shows the local accumulation of Cdc42 activity at invadopodia. We also include movies (Movie 6-7), to support this point.

1. Introduction, "FRET signal is typically measured with a wide field microscope.": This reviewer does not agree with this statement. Confocal and two-photon microscopes have also been used widely.

Fair point. We changed the text to "when the FRET signal is measured with a wide field microscope"

2. Introduction, "G-protein activating proteins (GAP)": It should read as "GTPase-activating proteins (GAPs)"

Thanks, corrected.

3. TRIF should read as TIRF.

All instances have been corrected.

4. Fig.1: To the best of this reviewer's knowledge, PKN1 was first used as the RhoA target peptide by Yoshizaki et al in 2003. J Cell Biol 162, 223-232. They also examined mDia, Rhoteki, and Rhophilin as the target peptides. Pak1 was first used as the Rac1 probe by Kraynov et al. Science 290, 333-337, 2000. Use of Pak1 as the Cdc42 probe was reported by Itoh et al. Mol Cell Biol 22, 6582-659, 2002. This reviewer believes that the priority of the first report should be respected.

We changed part of the introduction to:
High scoring proteins for interacting with constitutively active RhoA(Q63L) included ANLN part of the AniRBD Rho location sensor (Piekny and Glotzer, 2000), PKN1 part of aRho FRET sensor (Yoshizaki et al., 2003) and RTKN part of the rGBD Rho location sensor (Benink and Bement, 2005; Mahlandt et al., 2021) (Fig. 1A,B). This suggested that proteins with a high score in the mass spectrometry screen are potentially suitable as Rho GTPase activity biosensor. Indeed, the GBDs used for Cdc42 location sensors from, PAK1 used in the PBD location sensor (Itoh et al., 2002; Petrie et al., 2012) and N-WASP similar to WASp used in the wGBD location sensor (Benink and Bement, 2005) showed a high score in the screen (Fig. 1A,B).

Discussion:
Another challenge is the Rho GTPase specificity of the relocation-based sensor. For example, **Pak1(CRIB) was first used in a Rac1 FRET sensor (Kraynov et al., 2000).** Next, Pak1(CRIB) has been utilized in Cdc42 FRET sensors and in an intensiometric Cdc42 sensor (Hanna et al., 2014; Itoh et al., 2002; Kim et al., 2019). However, Pak1(CRIB), also named PBD sensor, has then been reintroduced by Weiner and colleagues as a Rac1 specific location-based sensor and is often used in neutrophil HL60 cells (Brunetti et al., 2022; Graziano et al., 2019; Le et al., 2021; Weiner et al., 2007).

We also updated the tables in Figure 1.

© 2023. Published by The Company of Biologists under the terms of the Creative Commons Attribution License (https://creativecommons.org/licenses/by/4.0/).

5. Fig. 1: Why do the authors omit other promising candidates shown in panel 1B? Please describe the reason for the choice.

We took into account the availability of plasmid DNA, as also explained in the manuscript: "candidate GBDs were selected from top 30 scores of the mass spectrometry screen, that were specific for one Rho GTPase and their DNA was available on addgene"

6. Fig. 1B: Be consistent to use either "Name" or "Uni Prot name" in Panel A.

We updated figure 1.

7. Fig. 2: Please include information on TOMM20. The readers may not read the paper by Gillingham et al.

We added an explanation: "To this end, a fusion with TOMM20 was used for mitochondrial localization."

8. Fig3 and 4: The authors should show the images of control H2A.
We provide the data for control H2A in figures 3B and 4B.

9. In Fig3B and 4B, "Cdc42/Rac1 affinity" would be misleading, because the control dots represent their authentic localization rather than "Cdc42/Rac1 affinity".

We agree, we have updated figure 3B and 4B.

10. Fig. 4: More explanation of this figure is required.

We added text: "Hence, the sensor candidate can freely partition between Rac and Cdc42 binding."

11. Fig. 5: More explanation about the FKBP-FRB system will be helpful.

We changed the text to: "The system used rapamycin induced heterodimerization of the two domains FRB and FKBP to recruit the DHPH domain of the Cdc42 specific GEF ITSN1 to the plasma membrane, where it induces activity of the endogenous Cdc42"

12. Fig. 6: It is rather surprising to see that control-mScarlet also responds to Rac1 activation. What is the explanation for this observation?

We agree and have no explanation.

13. Fig. 7: A single champion data may not be convincing to prove the usefulness of this probe.

We agree, we added another example to supplemental Figure 5. For the local accumulation of the Cdc42 activity near invadopodia we provide movies of three cells (Movie 5,6).

Reviewer #3

1) The discussion comparing different types of biosensors missed important points. Although the advantages of localization biosensors listed by the authors are correct, they gave the impression that these should simply be an improved replacement for FRET biosensors. There are times when FRET biosensors provide clear advantages. Unlike other proteins, Rho GTPases are well suited for localization sensors because the activated conformation, and only the activated conformation, localizes to the membrane. For diffuse or 3D localization FRET can provide better quantification. Subtle features such as gradients are not easily quantified over a background of unattached domain. The authors state that localization biosensors have enhanced spatial resolution, but this is not explained.

We agree that our introduction is biased towards a preference for relocation based biosensors. However, having used both approaches, we see that both strategies have pro's and cons.

© 2023. Published by The Company of Biologists under the terms of the Creative Commons Attribution License (https://creativecommons.org/licenses/by/4.0/).

Therefore, we removed the claim for higher resolution and we added: "Still, the ratiometric mode of imaging FRET sensors is beneficial for detection of gradients or activity in 3D imaging".

2) Throughout the paper, the ratio between the GTPase and the domain, and the overall expression level of each, was not sufficiently examined. The results in many cases would be dependent on both these factors (was a large excess of domain used? Was there insufficient domain to bind the GTPase and provide a signal? Did this vary for different domains, and therefore produce the differences observed? A lack of apparent binding specificity could be produced by high domain expression.)

We added the following:
The influence of the expression level of the nuclear constitutively active Cdc42, and its nuclear enrichment, as well as the expression level of the sensor candidate on the nuclear enrichment of the sensor candidate were tested for correlation and found neglectable (Supplemental Figure 2A,B,C).

© 2023. Published by The Company of Biologists under the terms of the Creative Commons Attribution License (https://creativecommons.org/licenses/by/4.0/).

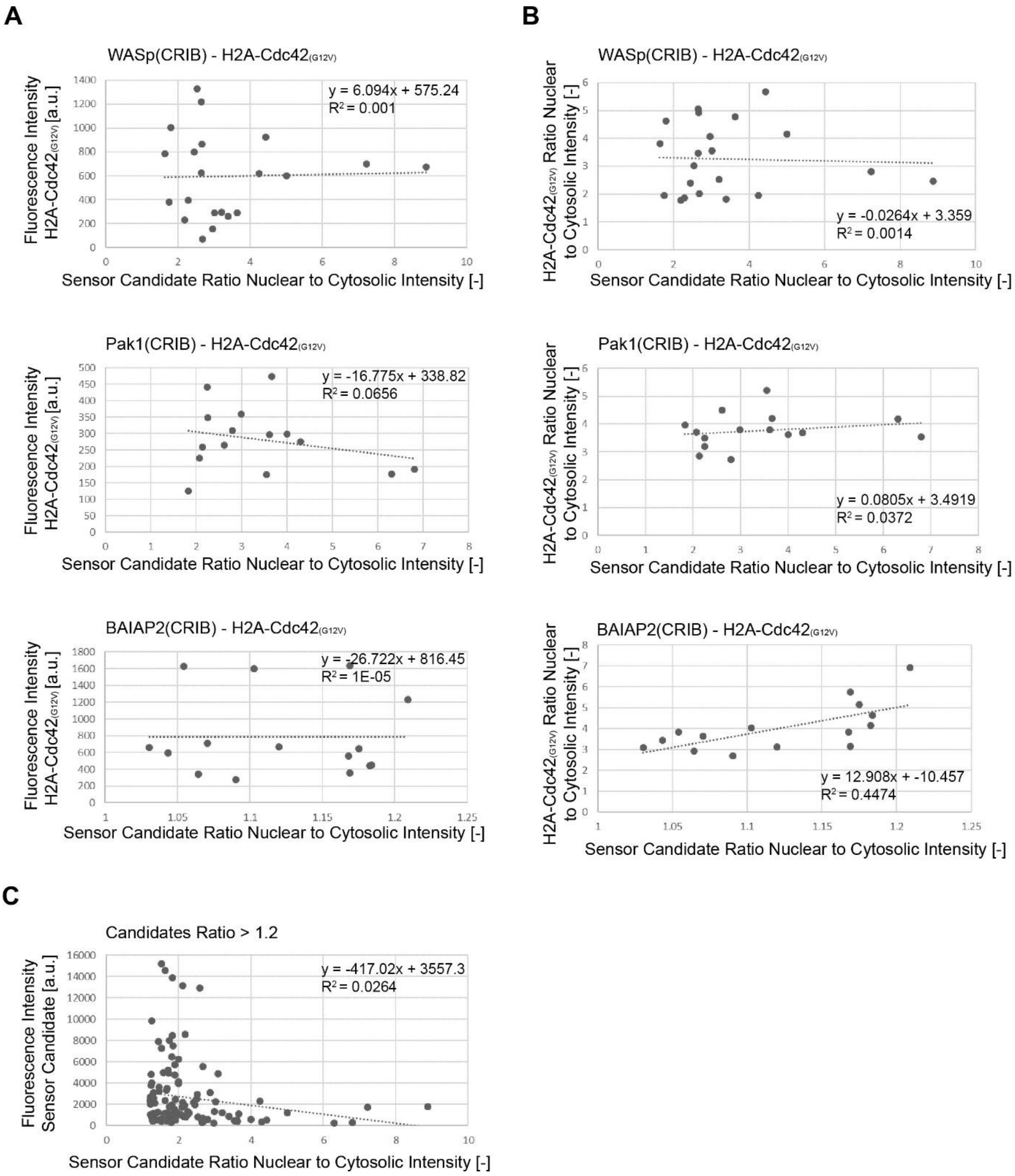

*Supplemental Figure 2. (A) Plots of the correlation between expression level of H2A-Cdc42(G12V)-ΔCaaX, represented by fluorescence intensity, and sensor candidate nuclear to cytosolic intensity, at the example of WASp(CRIB), Pak1(CRIB) and BAIAP2(CRIB), for the data shown in **Fig. 3B**. The general linear model (top, right corner) was fit to the data and is indicated as a dashed line. (**B**) Plots of the correlation between H2A-Cdc42(G12V)- ΔCaaX nuclear to cytosolic intensity and sensor candidate nuclear to cytosolic intensity, at the example of WASp(CRIB), Pak1(CRIB) and BAIAP2(CRIB), for the data shown in **Fig. 3B**. The general linear model (bottom, right corner) was fit to the data and is indicated as a dashed line. (C) Plots of the correlation between expression level of the sensor candidate, represented by fluorescence intensity, and sensor candidate nuclear to cytosolic intensity, for all candidates with a ratio above 1.2, for the data shown in **Fig. 3B**. The general linear model (top, right corner) was fit to the data and is indicated as a dashed line.*

© 2023. Published by The Company of Biologists under the terms of the Creative Commons Attribution License (https://creativecommons.org/licenses/by/4.0/).

3) In the nuclear exclusion assay, some GTPases were excluded from the nucleus and others not. This was true even without expression of the domains. When GTPases were excluded from the nucleus, domains were eliminated from contention, even when this was true without domain. The authors could at least mention that these domains may be viable.

Correct, and we have added this text: "we cannot exclude that these would be viable Cdc42 sensor candidates"

4) In the multiplexing experiment, only two cells were imaged. In one cell RhoA activity was inversely correlated with Cdc42 activity. In the other cell it was not. It seems there is insufficient information to reach firm conclusions.

We have added another example to Supplemental Fig. 5.

Minor points:

- There appear to be errors in naming mutants. Q60L is used for constitutively active Rac, but Q61L is likely meant. H2A-mTurquoise2-Rac1(G12V)-?CaaX is used when it likely should be H2A-mTurquoise2-Rac1(Q61L)-?CaaX. There are other examples -- a careful check of these names throughout the manuscript would be valuable.

Thanks for spotting this. Q60L is changed to Q61L. Note that the Rac1(G12V) is correct as it also is a constitutive active Rac1.

- Intro-Paragraph 1-line 5: change present to presence
- Intro-Paragraph 5- line 7: use them instead of theme.

Thanks, both were corrected.

Second decision letter

MS ID#: JOCES/2022/260802

MS TITLE: Cell-based optimisation and characterisation of genetically encoded, location-based biosensors for Cdc42 or Rac activity

AUTHORS: Eike K. Mahlandt, Gabriel Kreider-Letterman, Anna O. Chertkova, Rafael Garcia-Mata, and Joachim Goedhart

ARTICLE TYPE: Tools and Resources

Thank you for sending your manuscript to Journal of Cell Science through Review Commons.

I am happy to tell you that your manuscript has been accepted for publication in Journal of Cell Science, pending standard ethics checks. Where referee reports on this version are available, they are appended below.

© 2023. Published by The Company of Biologists under the terms of the Creative Commons Attribution License (https://creativecommons.org/licenses/by/4.0/).
